# Deletion of NoxO1 limits atherosclerosis development in female mice

**DOI:** 10.1016/j.redox.2020.101713

**Published:** 2020-09-04

**Authors:** Giulia K. Buchmann, Christoph Schürmann, Tim Warwick, Marcel H. Schulz, Manuela Spaeth, Oliver J. Müller, Katrin Schröder, Hanjoong Jo, Norbert Weissmann, Ralf P. Brandes

**Affiliations:** a Institute for Cardiovascular Physiology, Goethe-University, Theodor-Stern Kai 7, 60590, Frankfurt Am Main, Germany; b German Center for Cardiovascular Research (DZHK), Partner Site Rhein Main, Theodor-Stern Kai 7, 60590, Frankfurt Am Main, Germany; c Institute for Cardiovascular Regeneration, Goethe-University, Theodor-Stern Kai 7, 60590, Frankfurt Am Main, Germany; d Department of Internal Medicine III, University of Kiel, Arnold-Heller-Straße 3, 24105, Kiel, Germany; e German Center for Cardiovascular Research (DZHK), Partner Site Hamburg/Kiel/Lübeck, Arnold-Heller-Straße 3, 24105, Kiel, Germany; f Wallace H. Coulter Department of Biomedical Engineering, Georgia Institute of Technology and Emory University, 313 Ferst Dr NW, Atlanta, GA, 30332, USA; g Excellence Cluster Cardio-Pulmonary Institute (CPI), Universities of Giessen and Marburg Lung Center (UGMLC), Member of the German Center for Lung Research (DZL), Ludwigstraße 23, 35390, Gießen, Germany

**Keywords:** NADPH oxidase, NoxO1, Atherosclerosis, Gender differences, PCSK9, Reactive oxygen species, AAV, Adeno-associated virus, EC, Endothelial cells, LDL, Low-density lipoprotein, Lepr, Leptin receptor, MACEseq, Massive analysis of cDNA ends RNAseq, NoxO1, Nox organizer-1, PCSK9, pro-protein convertase subtilisin/kexin type 9, SMC, Smooth muscle cells, VEGF, Vascular endothelial growth factor, WT, Wildtype

## Abstract

**Objective:**

Oxidative stress is a risk factor for atherosclerosis. NADPH oxidases of the Nox family produce ROS but their contribution to atherosclerosis development is less clear. Nox2 promotes and Nox4 rather limits atherosclerosis. Although Nox1 with its cytosolic co-factors are largely expressed in epithelial cells, a role for Nox1 for atherosclerosis development was suggested. To further define the role of this homologue, the role of its essential cytosolic cofactor, NoxO1, was determined for atherosclerosis development with the aid of knockout mice.

**Methods and results:**

Wildtype (WT) and NoxO1 knockout mice were treated with high fat diet and adeno-associated virus (AAV) overexpressing pro-protein convertase subtilisin/kexin type 9 (PCSK9) to induce hepatic low-density lipoprotein (LDL) receptor loss. As a result, massive hypercholesterolemia was induced and spontaneous atherosclerosis developed within three month. Deletion of NoxO1 reduced atherosclerosis formation in brachiocephalic artery and aortic arch in female but not male NoxO1−/− mice as compared to WT littermates. This was associated with a reduced pro-inflammatory cytokine signature in the plasma of female but not male NoxO1−/− mice. MACE-RNAseq of the vessel did not reveal this signature and the expression of the Nox1/NoxO1 system was low to not detectable.

**Conclusions:**

The scaffolding protein NoxO1 plays some role in atherosclerosis development in female mice probably by attenuating the global inflammatory burden.

## Highlights

• Reactive oxygen species (ROS) are thought to promote atherosclerosis.
• NoxO1 is a factor required for the activation of the ROS-producing Nox1.
• Deletion of NoxO1 attenuated atherosclerosis development in female mice.
• NoxO1 knockout suppressed inflammatory signatures in the female murine plasma.

## Graphical abstract

**Figure:**
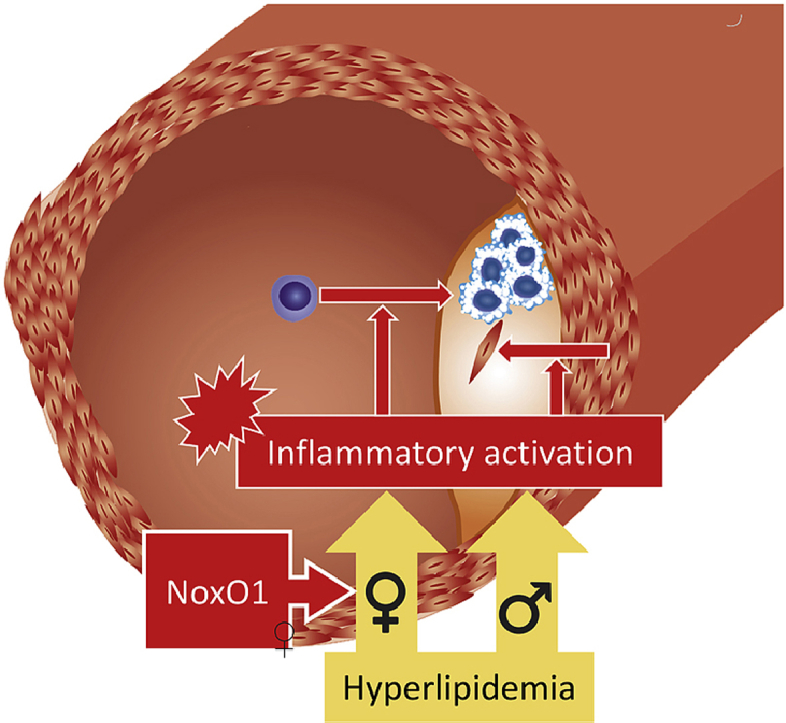

## 1 Introduction

NADPH oxidases of the Nox family are a main source of reactive oxygen species (ROS) in the cardiovascular system. ROS are important signaling molecules that modulate activity of proteins and maintain the cellular redox status [1]. Nox enzyme-derived ROS have been implicated in cardiovascular disease, however different functions have been attributed to the individual homologues. In the vascular system the NADPH oxidases Nox1, Nox2, Nox4 and Nox5 are expressed [[2], [3], [4]]. Vascular Nox1, Nox2 and Nox5 produce superoxide anions in response to agonist stimulation leading to inactivation of vasoprotective nitric oxide (NO) [5] and peroxynitrite formation. Nox4 is constitutively active in vascular endothelial cells (EC), smooth muscle cells (SMC) and adventitial fibroblasts [2] and produces hydrogen peroxide [6]. As hydrogen peroxide does not inactivate NO and may even substitute for it, Nox4 appears to have a protective function in the vessel [7].

Different to Nox4, the homologues Nox1 and Nox2 require further cytosolic subunits to form an activated ROS-producing complex. The cytosolic subunits p67phox or NoxA1 are needed to activate the complex, p47phox or NoxO1 function as scaffolding proteins between the membrane-bound Nox subunit and the activator. Nox organizer-1 (NoxO1) is a functional homologue to p47phox, but lacks the autoinhibitory subunit resulting in high basal activity [8]. NoxO1 is highly expressed in testis and colon [9], moreover also vasculatur expression was reported [10,11]. The function of NoxO1 in the vasculature, however, is not fully understood. Studies on NoxO1 knockout mice suggested that its deletion leads to decreased superoxide anion production and increased angiogenesis [10]. In diabetic mice, NoxO1 deletion induced an anti-inflammatory phenotype, leading to the hypothesis of NoxO1 as driver of vascular dysfunction [11].

Although the mouse is the preferred laboratory animal, rodents do not develop spontaneous atherosclerosis. Most studies therefore employ a combination of genetic deficiency of either ApoE or LDL-receptor together with high fat feeding to induced plaque formation within a few month. These models are characterized by excessive lipid levels and high inflammatory activation. The need to cross the animals into either the ApoE or LDL-receptor knockout background further complicates the studies and the heterogeneity of the readouts.

Recently, a technique to induce hypercholesterolemia in mice by an adeno-associated virus (AAV) mediated overexpression of the pro-protein convertase subtilisin/kexin type 9 (PCSK9) was developed [12]. PCSK9 binds hepatic low-density lipoprotein (LDL) receptors and directs them for degradation in lysosomes. Overexpression of PCSK9 results in elevated LDL levels. A single injection of AAV-PCSK9 in combination with a high fat diet is sufficient to induce spontaneous atherosclerotic lesions after 3 month [13]. This technique therefore not only eliminates the need for crossing into a hyperlipidemic background, it also provides a time-saving approach to induce atherosclerosis.

Along the classic concept of ROS-mediated damage and inflammation, NADPH oxidases of the Nox family are thought to promote atherosclerotic development. This function is best documented for Nox2, which is the predominant Nox homologue in leukocytes [9]. Lipid peroxidation by ROS-derived of Nox enzymes results in initiation of inflammatory signaling, which is an early event in atherosclerotic plaque development [14]. Despite a convincing conceptual basis, the data to support a strong role of Nox enzymes for atherosclerosis development in mouse models are rather weak and conflicting. Whereas Nox4 even has a clear anti-atherosclerotic function [7,15,16], most, but not all studies suggest that the Nox2 complex is pro-atherosclerotic [[17], [18], [19], [20]]. The situation for Nox1 is less clear. It appears that the enzyme modulates atherosclerosis development, but does not significantly attenuate the process [[20], [21], [22], [23]]. An alternative approach to dissect the function of Nox1 is studying its cytosolic activators. One study suggests that deletion of NoxA1 attenuates plaque development but the function of NoxO1 is still unknown [24]. Here we set out to determine the role of NoxO1 for atherosclerosis development in mice using the AAV-PCSK9 overexpression system combined with a high fat diet.

## 2 Material and methods

### 2.1 Mouse studies

Animal complied with the ARRIVE guidelines and were carried out in accordance with the EU Directive 2010/63/EU for animal experiments. The University Animal Care Committee and the Federal Authorities for Animal Research (Darmstadt, Germany) approved the study protocol (approval number: FU1095, FU1214). Male and female mice were used in this study.

Global knockout mice for NoxO1 were generated as previously described and bred in a heterozygous fashion, to obtain ideal genetic wildtype controls for the knockout littermates [10]. Mice were housed in a specified pathogen-free facility with 12/12 h day and night cycle and free access to water and chow every time.

### 2.2 AAV vector production and purification

AAV serotype 8 vectors for expression of the murine D377Y-PCSK9 cDNA (AAV-PCSK9) or CMV-luciferase (AAV-Luc) were produced using the two-plasmid-method by co-transfecting AAV/D377Y-mPCSK9 (gift from Jacob Bentzon; Addgene plasmid # 58376) [12] or pSSV9-CMV-Luc together with the helper plasmid pDP8 [25] in HEK293T cells using polyethylenimine (Sigma Aldrich). AAV vectors were purified using iodixanol step gradients and titrated as previously described [26].

### 2.3 Bioluminescence measurements

For bioluminescence studies, mice were injected intravenously by tail vein injections with AAV-Luc (1.0 × 10^11^ VG/mouse) or sodium chloride. Bioluminescence was analyzed 7 days later using the Fusion FX7 (Vilber). Mice were anesthetized and injected intraperitoneal with 200 μl luciferin (15 mg/ml, Caliper LifeSciences). Images were acquired 10 min after luciferin application.

### 2.4 Blood measurements

Blood samples were collected from the heart after sacrificing. Samples were kept at room temperature for 30min, centrifuged for 10 min at 800 g and serum was snap frozen in liquid nitrogen. Lipid profiles were determined in serum samples from mice using a Cobas 8000 Modul C 701 system (Roche). A PCSK9-specific ELISA was conducted to measure the level of endogenous mPCSK9 (#DY3985, R&D Systems).

### 2.5 Immunoblotting

Pulverized liver tissue samples were lysed using the following lysis buffer (pH 7.4): Tris-HCl (50 mmol/L), NaCl (150 mmol/L), sodium pyrophosphate (10 mmol/L), sodium fluoride (20 mmol/L), nonidet P40 (1%), sodium desoxycholate (0.5%), proteinase inhibitor mix, phenylmethylsulfonyl fluoride (1 mmol/L), orthovanadate (2 mmol/L), okadaic acid (0.00001 mmol/L). Samples were cooked in sample buffer and separated by SDS-PAGE followed by western blotting. Western blot analyses were performed with an infrared-based detection system (Odyssey, Licor). LDL-R primary antibodies were obtained from BioVision (1:200, #3839-100, BioVision), anti-β-actin antibodies from Sigma (1:1000, #A1978, Sigma). Conjugated secondary antibodies, obtained from Licor (1:10000, #926–68073/#926–32212), were used for IR-fluorescence-based detection of the proteins.

### 2.6 Spontaneous atherosclerosis model & AAV-mPCSK9 application

Mice were injected with AAV-PCSK9 (1 × 10^11^ VG), AAV-Luc (1 × 10^11^ VG) or saline via tail vein at an age of 10 weeks. Afterwards, mice were fed a special PAIGEN high-fat diet (EF PAIGEN 1.25% cholesterol, 0,5% Na-Cholate, Ssniff, Soest, Germany) for three months and subsequently sacrificed. The aortic arch, aorta, heart, blood, liver and carotids were collected and macroscopic images of the aortas were acquired. For molecular histology studies, carotid artery and aortic arches were embedded in OCT compound and stored at −80 °C. Thoracic and abdominal aortas were cleaned, snap frozen and stored at −80 °C. The heart was fixed with 4% PFA overnight, dehydrated in 30% sucrose overnight and embedded in OCT compound.

### 2.7 Histology

Carotid tissue samples frozen in OCT compound were prepared as 10 μm serial sections. Sections were stained with Oil-Red-O dye. The brachiocephalic arteries were analyzed for plaque area by planimetry with ImageJ. The heart was sectioned consecutively (10 μm sections) starting from the commissures of the aortic cusps upwards. Sections were stained with Oil-Red-O. The aortic root lesion area was determined using image analysis software (ImageJ).

### 2.8 Macroscopic plaque determination

The aortas were cleaned of connective tissue and photographed using a Nikon DS-Fi2 camera. Vessel surface area and plaque surface area were determined using the ImageJ program in a blinded fashion, and relative plaque area was calculated.

### 2.9 MACEseq and bioinformatics

For Massive analysis of cDNA ends RNAseq (MACEseq) 50 ng RNA obtained from abdominal aorta from one individual animal was used. Three to five different animals of each sex were analyzed from the WT and NoxO1−/− group. The library preparation was conducted by GenXPro. 3'mRNA libraries were prepared using GenXPros “Rapid MACE-Seq Kit” for low-input mRNA according to the manual of the manufacturers. Briefly, RNA was fragmented to an average size of 350 bps, followed by poly-A specific cDNA synthesis, pooling and amplification by PCR using the minimum number of cycles as described in the manual. The PCR product was purified by SPRI purification. The final product of 200–400 bps was quality controlled on a PerkinElmer LabChip GXII, the concentration was measured on Qbit. Sequencing was performed on an Illumina NextSeq 500 machine with 1 × 75 bps. The MACE-reads were demultiplexed according to the sample IDs, PCR duplicates were removed with the help of the “TrueQuant” UMI barcodes. Low-quality and adapter-containing reads were cropped. The reads were mapped to the mouse genome (GRCm38. p6) and genes were quantified for the individual libraries for gene expression. Differential gene expression of the different pairwise comparisons was analyzed using DESeq2 [27]. The normalized gene counts, p-values and log2fold-change values of all pairwise comparisons were combined in a single table. Differentially expressed genes were used to calculate GO enrichment using the hypergeometric distribution using the GenXPro analysis pipeline and web-service. Co-expression analysis of the MACEseq data was conducted using the WGCNA R-package [28]. All expression samples from animals for which cytokine assays were measured were used for the analysis to allow integration (see below). The expression data was rlog transformed using DEseq2 and genes with a MAD >0.1 and median expression >0 over all samples, were retained for correlation analysis. The following parameters for the module creation with the WGCNA function blockwiseModules were used (power = 19, TOMType = “signed”, minModuleSize = 30,reassignThreshold = 0, mergeCutHeight = 0.3). A soft-thresholding power of 19 was choosen after inspection of a range of values as suggested in the WGCNA manual. Cytokine measurements were correlated with mean expression values of all WGCNA co-expression modules. Significant cytokine-module relationships were defined as modules that had an absolute correlation of at least 0.8 (p-value <0.001) with cytokine abundance.

### 2.10 Cytokine array

Serum samples were analyzed with the commercial cytokine array AAM-CYT-G3 (RayBiotech, Norcross GA). Hydration and scanning of the array was performed by Tebu-bio according to the manufacturer's instructions. In brief, membranes were blocked for 30 min with blocking buffer, incubated with serum samples (diluted with blocking buffer) and scanned.

### 2.11 Statistics

Unless otherwise indicated, data are given as means ± standard error of mean (SEM). n indicates the number of individual experiments or animals. Calculations were performed with Prism 8.0. The latter was also used to test for normal distribution and similarity of variance. Individual statistics of unpaired samples was performed by *t*-test, Mann-Whitney test or ANOVA followed by post-hoc testing, according to normal distribution and group number. A p-value of <0.05 was considered as significant. Correction for multiple testing was performed according to Bonferroni (padj), except for sequencing data, which was corrected according to Benjamini Hochberg (FDR).

## 3 Results

### 3.1 AAV-PCSK9 induces hypercholesterolemia

**Fig. 1:**
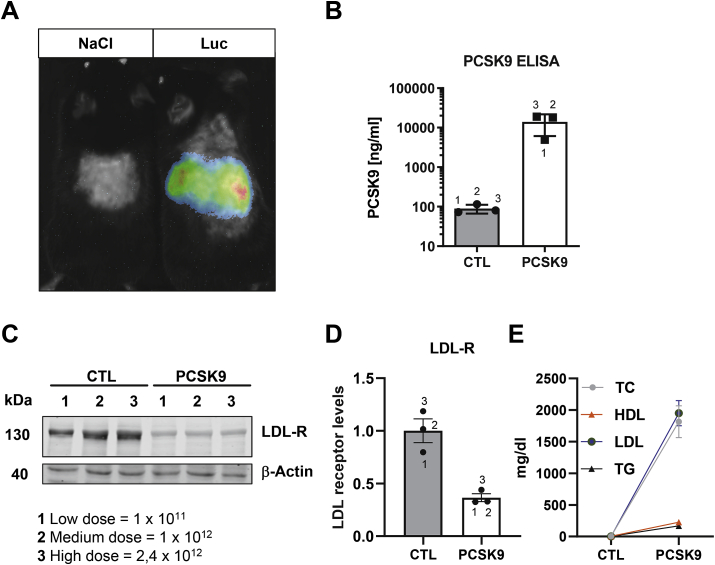
**AAV-PCSK9 induces hypercholesterolemia**. **A** Bioluminescence imaging of mice one week after a single injection of AAV-Luc or saline. **B** Serum mPCSK9 level (1: Low dose = 1 × 10^11^; 2: Medium dose = 1 × 10^12^; 3: High dose = 2,4 × 10^12^) (mean ± SEM) 4 weeks after injection of AAV-PCSK9 or saline (CTL). **C and D** Hepatic LDL-Receptor expression normalized to β-Actin (n = 3; mean ± SEM) 4 weeks after injection **E** Total plasma cholesterol (TC), high density lipoprotein (HDL), low density lipoprotein (LDL) and triglyceride (TG) serum levels (n = 3–9) 4 weeks after injection of mice treated with chow diet; (mean ± SEM).

In order to induce atherosclerosis development we used the AAV-PCSK9 system to overexpress PCSK9 stable in the liver. PCSK9 overexpression causes loss of hepatic LDL receptor surface expression and leads to hypercholesterolemia [12,13]. *In vivo* bioluminescence measurements of mice treated with AAV-Luc confirmed a liver-specific expression of our constructs (Fig. 1A). The successful overexpression of PCSK9 was evident by the massive increase in PCSK9 plasma levels (Fig. 1B), LDL-R protein expression (Fig. 1C and D) and plasma lipid content (Fig. 1E). LDL receptor expression was reduced by approximately 65% compared to AAV9-Luc control (Fig. 1C and D). Most importantly, PCSK9 overexpression induced hypercholesterolemia, as detected 4 weeks after PCSK9 injection (Fig. 1E) (total cholesterol ~1800 mg/dl). These results prove AAV-PCSK9 as effective system to mediate hypercholesterolemia in mice.

**Fig. 2:**
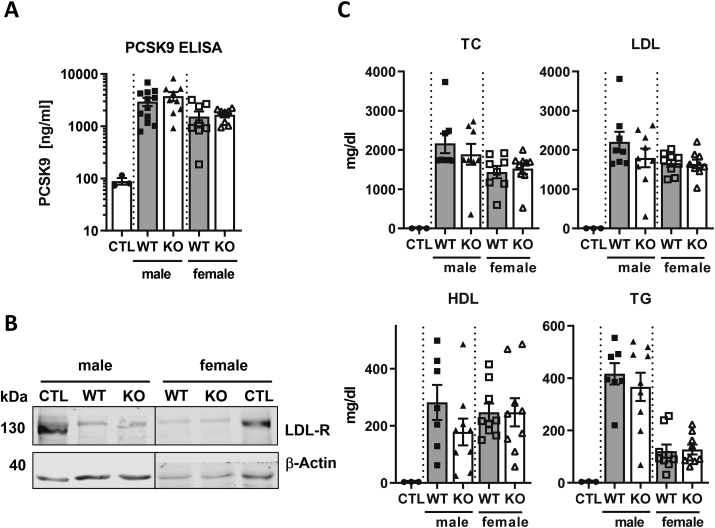
**Successful LDL-R degradation by PCSK9 overexpression**. NoxO1 knockout (KO) and wildtype (WT) mice treated with a single injection of AAV-PCSK9 (1 × 10^11^ VG) and 3 month Paigen diet (CTL = 1 × 10^11^ VG AAV-Luc). **A** Serum PCSK9 levels (n = 3–12; mean ± SEM). **B** Western blot analysis of LDL receptor and β-Actin of liver tissue samples. **C** Total Cholesterol (TC), HDL, LDL and triglyceride (TG) serum levels of male and female wildtype and NoxO1 KO mice (n = 7–9; mean ± SEM).

Subsequently we tested whether a single injection of AAV-PCSK9 induces comparable hypercholesterolemia between the mouse strains and genders. mPCSK9 levels in plasma samples were significantly increased in NoxO1 knockout and wildtype mice (Fig. 2A). Knockout of NoxO1 had no effect on lipid level, PCSK9 or LDL receptor expression (Fig. 2B and C). With respect to gender differences, it was observed that triglyceride levels were lower in females (male: ~400 mg/dl; female ~120 mg/dl), which is a known effect [29]. Collectively, these data suggest that the approach chosen here represents a valid model to induce prolonged massive hyperlipidemia in mice.

### 3.2 Deletion of NoxO1 reduces atherosclerotic lesions in female mice

**Fig. 3:**
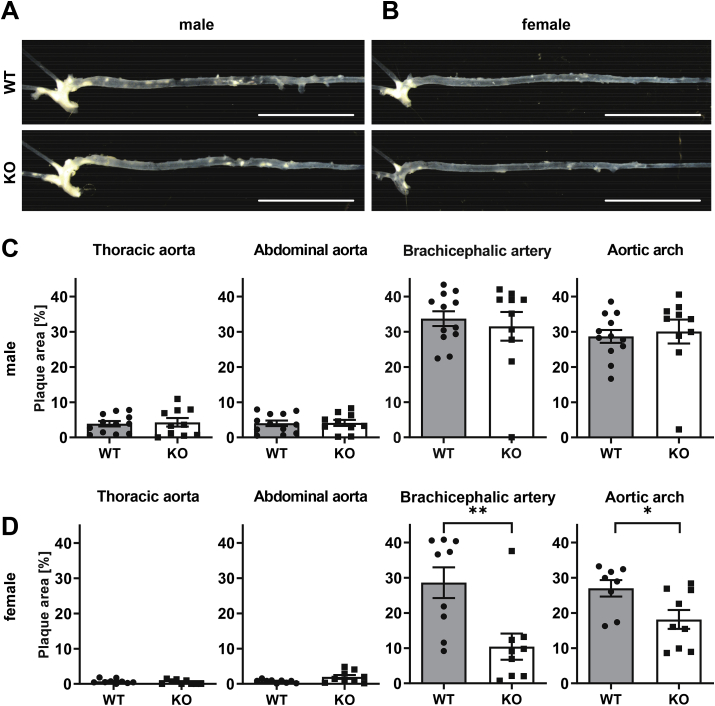
**Knockout of NoxO1 reduces atherosclerotic lesions in female mice**. NoxO1 knockout (KO) and wildtype (WT) mice treated with AAV-PCSK9 and 3 month Paigen diet. **A&B** Macroscopic overview of male (**A**) and female (**B**) wildtype and NoxO1 knockout aortas (scale bars 10 mm). **C&D** Quantified plaque area of male (**C**) and female (**D**) thoracic aorta, abdominal aorta, aortic arch and brachiocephalic artery in wildtype and NoxO1 knockout mice (n = 9–12; mean ± SEM; Mann-Whitney test or *t*-test; *p ≤ 0.05; **p ≤ 0.01).

**Fig. 4:**
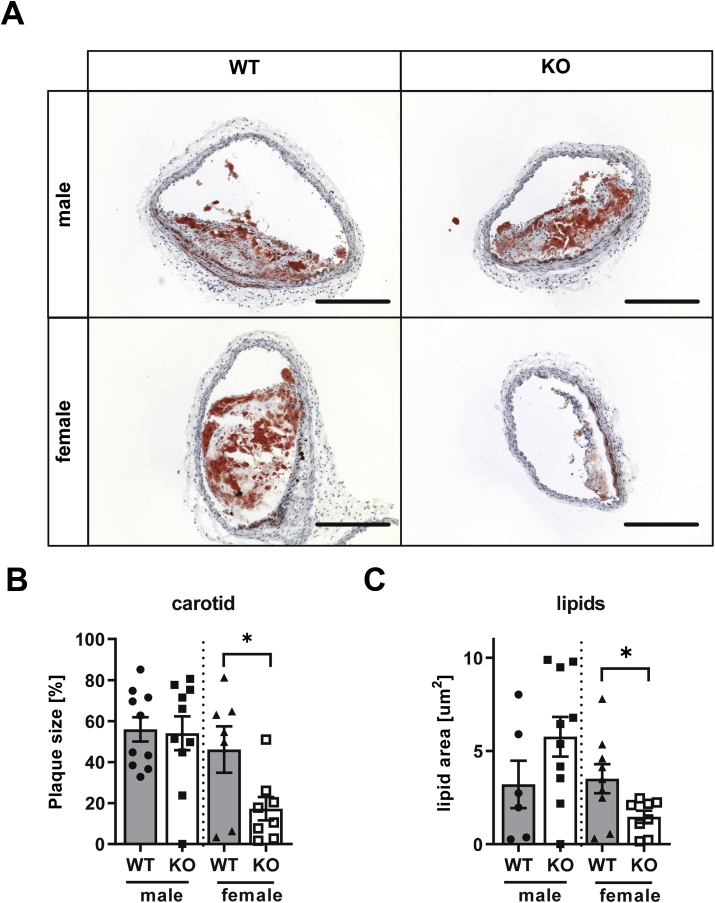
**Reduced plaque development in female NoxO1 KO mice in brachiocephalic artery**. NoxO1 knockout (KO) and wildtype (WT) mice treated with AAV-PCSK9 and 3 month Paigen diet. **A** Carotid plaque area of male and female wildtype and NoxO1 knockout mice (scale bars 500 μm). **B** Quantified carotid plaque areas (n = 7–10; mean ± SEM; *t*-test; *≤0,05). **C** Quantified lipid areas (n = 6–10; mean ± SEM; *t*-test; *≤0.05).

To investigate a contribution of NoxO1 in the development of atherosclerosis, plaque development was determined in wildtype and NoxO1 knockout littermates of both genders three months after AAV-PCSK9 injection followed by subsequent high-fat diet. After this time, plaque developed was evident, especially in areas of disturbed flow, like the aortic arch and the brachiocephalic artery (Fig. 3A and B). Deletion of NoxO1 had no effect on plaque development in male mice (Fig. 3A and C). In contrast to this, plaque size in the brachiocephalic artery and aortic arch of female NoxO1−/− was about 50% lower compared to their female littermate WT control (Fig. 3B and D). Oil-Red-O staining of frozen sections confirmed less plaque load in female, but not male NoxO1 knockouts, compared to WT controls (Fig. 4A and B). The plaque morphology was comparable in all conditions, but lipid content was reduced in samples with low plaque development (Fig. 4C). Knockout of NoxO1 in each sample was determined by qRT-PCR to confirm that the outliers had the correct genotype (Supp. 1) According to general practice, also plaque development at the aortic sinus was determined (Supp. 2). Massive plaque development was found in this area, which was probably too advanced to reflect a sensitive window. Despite this, there was a small increase in plaque size in NoxO1−/− mice of both genders, which reached the significance level in male mice (Supp. 2).

### 3.3 MACEseq does not identify relevant gene signatures

**Fig. 5:**
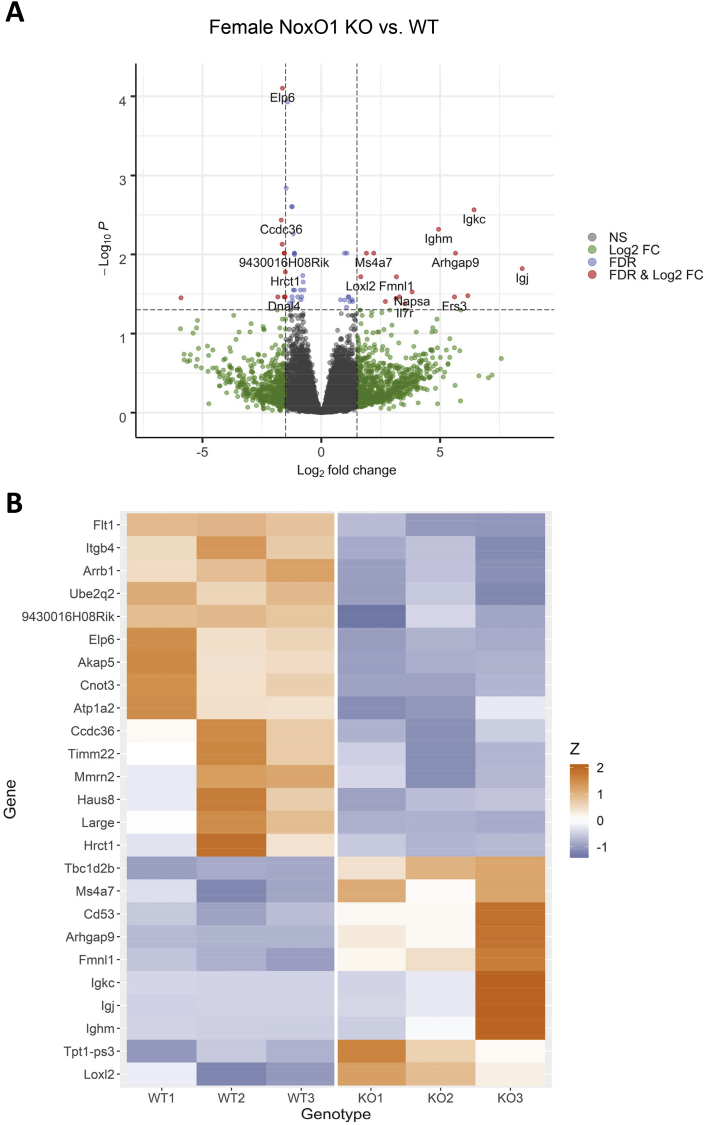
**Vascular gene expression in female aorta determined by MACEseq**. MACEseq dataset of wildtype and NoxO1 knockout abdominal aorta samples after AAV-PCSK9 treatment and 3 month Paigen diet. **A** Volcano blot of female MACEseq comparing KO versus WT (FDR = 0.05). **B** Heatmap of 25 most significant genes of female NoxO1 knockout compared to wildtype (n = 3).

To obtain insights into the molecular mechanisms resulting in differential atherosclerosis development, RNA sequencing was performed by MACEseq. To avoid that this readout was influenced by a different plaque size or composition, the abdominal aorta was used, assuming that the delayed progression in this area would eventually result in a similar plaque formation as in the aortic arch. Principal component analysis indicated that gene expression between male WT and male NoxO1−/− mice was very similar and that the gender of the animals had a stronger impact on gene expression than the NoxO1 expression (Supp. 3). Comparison of male NoxO1 knockout and wildtype animals identified no significant differentially regulated genes (FDR<0.05). Comparison of gene expression between female NoxO1 knockout and wildtype animals, indicated 56 significant regulated genes (FDR<0.05, Fig. 5A and Supplementary table 3.1). The 25 top significant genes are shown in Fig. 5B. KEGG analysis of DEGs identified no significant pathway regulation. GO Term analysis (p-value<0.05) revealed a slight tendency of lymphocyte related proliferation and regulation in female NoxO1 knockout mice (Supp 4 and Supp Table 3.3). Syk, Ptprc and Lat 2 are involved in B cell activation. Syk, Ptprc, Ccl2, Rasal3 and Ighm play a role in lymphocyte activation. There was also a loss in Flt 1 (VEGF-R1) and Itgb4 expression upon NoxO1 knockout which may suggest small alterations in endothelial gene expression or a reduced ratio of endothelial to other cells. Overall, the data speak for only small aortic gene expression differences between NoxO1 knockout and WT mice. This may suggest that the main site of action of NoxO1 is not the vascular wall or a vascular cell, which contributes little to overall gene expression.

### 3.4 Antioxidant and oxidant pathways are not impaired by NoxO1 deletion

We previously reported that there is a slight reduction in the vascular ROS production of NoxO1−/− mice [11]. In order to differentiate this between genders and atherosclerosis, the expression of pro- and antioxidant genes was compared from the MACEseq data set: Oxidases such as Noxes, cytochrome c oxidases as well as xanthine oxidases were not altered by NoxO1 deletion (Supp.6 and Supp. 7). Gene expression of antioxidant scavenger enzymes such as superoxide dismutase (SOD), catalase (CAT), thioredoxin (TRX) and peroxiredoxin (PRX) remained unchanged (Supp. 6 and Supp. 7). Collectively, also these data speak against pronounced redox alterations in response to NoxO1 deletion in the vessel wall and further support the overall gene expression data: It appears that not vessel intrinsic functions are responsible for the protection of female NoxO1−/− mice against atherosclerosis development.

### 3.5 Cytokine array indicates a significant anti-inflammatory response in female NoxO1−/− mice

**Fig. 6:**
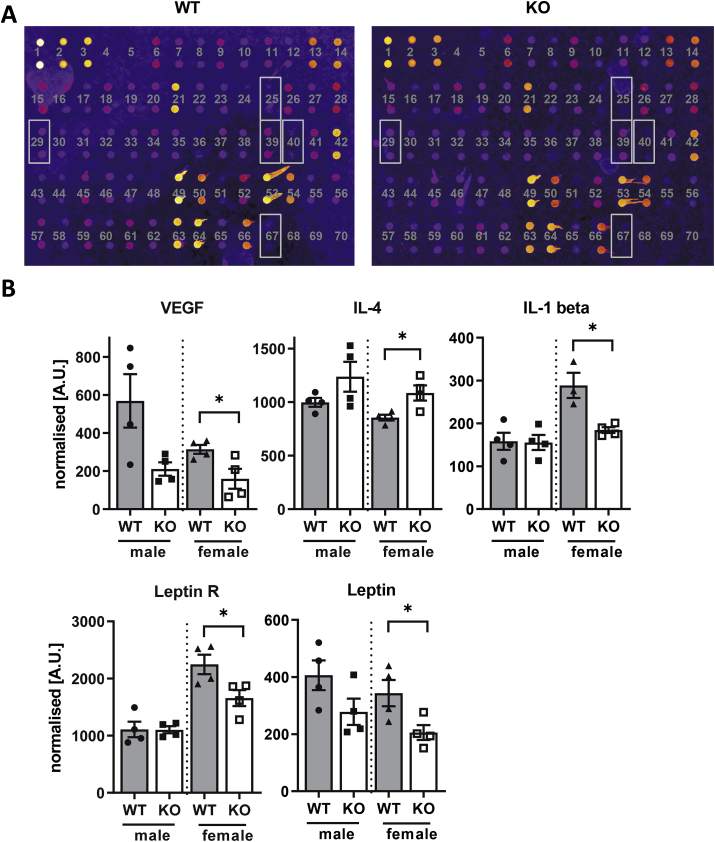
**As compared to female WT mice, the plasma of female NoxO1 KO mice has a more anti-inflammatory profile**. NoxO1 knockout (KO) and wildtype (WT) mice treated with AAV-PCSK9 and 3 month Paigen diet. **A and B** Plasma antibody array (AAM-CYT-G3 RayBiotech) of wildtype and NoxO1 knockout mice serum samples (n = 4; mean ± SEM, *t*-test, *p ≤ 0.05 WT vs. KO).

The similarity in vascular gene expression may suggest that the difference in atherosclerosis is more a reflection of systemic inflammatory changes. To study this, plasma cytokines were measured which should provide a better reflection of whole body inflammatory activity. Serum samples of female NoxO1 knockout mice contained less IL-1β and more IL-4 than their WT female littermates (Fig. 6 and Supp. 8). This suggests an anti-inflammatory situation in female NoxO1 knockout mice. Moreover, female but not male NoxO1−/− mice exhibited a significant reduction of the leptin receptor, leptin and VEGF. VEGF is known to be induced by ROS and to promote atherosclerosis [30] and leptin is an adipokine and has been shown to stimulate the immune responses [31] and to increase sympathetic drive [32]. The extent of atherosclerosis and severity is associated with leptin levels [33,34]. Lower contents of leptin and leptin receptor in female NoxO1 knockout samples indicate an anti-atherosclerotic effect of NoxO1 deletion. Collectively, these data suggest that a systemic attenuation of the inflammatory response in female NoxO1−/− mice contributes to the protection from atherosclerosis development. In order to substantiate this finding, it was tested by correlation analysis, whether components significantly altered in the mouse serum interact with vascular gene expression. We obtained 74 gene co-expression modules from the MACEseq data. Indeed, we found that four modules were in part positively or negatively correlated with the significantly altered plasma cytokines (Figure Supp 9). Supp excel Tables 1.1–1.4 lists the genes contained in those four modules. Supp excel Tables 2.1–2.4 reports overrepresented GO terms, KEGG pathways and enriched transcription factor motifs. Interestingly, high plasma leptin receptor expression correlated with genes associated with pathways indicative for oxidoreductase activity and electron transport chain (module 14) and IRF4 (module 15). Although none of the pathways suggested direct follow-up, the analysis supports the notion that vascular gene expression in the present model is strongly affected by plasma cytokines.

### 3.6 Gender differences in NADPH oxidase expression

Principal component analysis of MACEseq data revealed a strong impact of gender on gene expression (Supp. 3). Atherosclerosis development between NoxO1 knockout and wildtype was significantly changed in females only (Figs. 3 and 4). Because of these gender specific differences, we decided to determine changes in gene expression of the redox system between genders. Comparison between male and female wildtypes resulted in 944 significantly differentially expressed genes (FDR≤0.05) (Supp. 10 and Supp. Excel Tables 3.2); 450 upregulated and 494 downregulated genes comparing female and male. We found significant expression changes in the oxidase Nox2, antioxidative Glrx and ApoE between female and male (Table 1). The genes Eif2s3y, Ddx3y, Kdm5d and Xist are known to be differentially expressed between genders [35] and served as positive control (Table 1 and Supp. 10).

**Table 1:** Gender specific gene expression in aorta and liver tissue. Gene expression of redox and gender specific genes comparing female against male wildtypes in abdominal aorta and liver tissue.

Genes		Present study		Lau-Corona et al.		Abu-Toamih et al.	
		Abdominal aorta		Hepatic tissue		Hepatic tissue	
		log 2	FDR	log 2	FDR	log 2	FDR
redox genes	Glrx	−1,06	4,14E-02	−1,37	3,74E-05		
	Sod 3	−0,61	8,45E-02	1,30	9,64E-03		
	Cat	0,84	6,58E-02	−1,28	1,91E-03		
	Gsta2	−0,96	NA	−1,73	5,93E-03		
	Gstk1	−0,85	5,25E-02	1,33	4,97E-03		
	Gstm1	0,02	9,75E-01	−1,32	6,38E-03		
	Gstm3	−2,84	NA	−1,51	1,90E-07		
	Gstp2	−0,10	8,76E-01	−12,15	2,00E-26		
	Gstp1	−0,11	8,47E-01	−7,99	3,27E-148		
	Gstt1	−0,61	5,26E-02	1,72	2,76E-10		
	Gstt3	0,24	7,83E-01	4,75	2,41E-57		
	Cyba	−1,68	8,72E-10				
	Cybb	−1,77	6,98E-05	1,74	2,96E-04		
	Nox4	−0,09	8,98E-01	−6,78	5,65E-78	−1,48	1,00E-02
	Xdh	0,46	5,06E-01				
gender genes	Eif2s3y	−4,94	1,93E-07				
	Xist	9,64	2,03E-20				
	Ddx3y	−6,74	2,81E-19	−304,84	0,00E+00		
	Kdm5d	−3,91	5,21E-03	−487,54	1,07E-170	−2,34	1,00E-02
other	Lepr	0,48	4,31E-01			1,55	1,00E-02
	Apoe	−1,22	5,07E-04				

To validate the data regarding gender differences we examined two studies about gender differences in hepatic tissue. A study of Lau-Corona et al. comparing gender specific effects in liver tissue [36,37] showed sex specific differences in glutathion-S-transferases, Nox2 and Nox4 (Table 1). Nox4 was shown to be downregulated in females. Another liver specific study of Abu-Toamih confirmed downregulation of Nox4 in females [37] (Table 1). This study additionally described higher Lepr expression in female mice. Cytokine array results of serum samples of the present study make a similar observation (Fig. 6).

## 4 Discussion

In this study, we observed that global deletion of NoxO1 limits spontaneous atherosclerotic development in female mice. Loss of NoxO1 resulted in reduced plaque formation and lipid accumulation in the brachiocephalic artery and aortic arch, whereas the distal aortic regions carried few plaques in total. It is known that atherosclerosis development in mice starts proximal, at the aortic sinus and spreads to the distal aorta and that plaques occur prevalently in areas with disturbed blood flow [38]. Thus, it was not surprising, that the aortic roots plaque burden was so severe that the protective effect of NoxO1 deletion was no longer seen.

The anti-atherosclerotic effect of NoxO1 deletion was restricted to female mice, which was an unexpected finding. Vascular reactivity is known to be different between male and female rodents [39], which however, largely relates to the contractile response [[40], [41], [42], [43]], a differential expression of cytochrome P450 monooxygenases and the production of vasoactive lipids [44]. Moreover, some studies [[45], [46], [47]], even long ago from our lab [48] suggested that male rodents exhibit a greater lucigenin chemiluminescence signal, which was taken as readout for superoxide by that time and attributed to NADPH oxidases activity [[49], [50], [51]] and antioxidant enzymes [52]. Little is known on gender specific Nox expression. Aortic MACEseq of the present study revealed a reduced expression of Nox2, p22phox and p67phox in female mice but other NADPH oxidase family members were either not detected or not differentially expressed. It is, however, uncertain whether this reflects differences in vascular leukocyte trapping or a true effect of gender on Nox expression.

On the aortic RNA level, we could not identify a strong impact of NoxO1 on gene expression, whereas in the serum, several cytokines were differentially regulated upon NoxO1 deletion, suggesting an attenuated degree of inflammatory activity. That this might indeed be the case, is supported by a previous study from our group, which observed a similar phenotype in a mouse diabetes model [11]. Despite this, the overall importance of NoxO1 for atherosclerosis development appears to be limited. Comparison of the cytokine array with the RNAseq data also points towards the possibility that hepatic or myeloid but not vascular NoxO1 might be of relevance for the present study. This appears possible as a global knockout mouse was used in the present study, of which we know that the lifespan is expanded [53] and that inflammatory burden is reduced [11]. Moreover, it has been shown that NoxO1, potentially through Nox1, promotes peroxynitrite formation, which contributes to the development of degenerative diseases like COPD [54].

As mentioned in the introduction, a careful analysis on the evidence of a contribution of Nox enzymes to atherosclerosis development yields rather ambiguous findings. While the specific function of NoxO1 has not been studied, the contribution of Nox1 was analyzed: Nox1 deletion attenuated lesion development in the partial carotid artery ligation model [13,23]. Whereas two studies suggest that ApoE/Nox1 double knockout mice developed less atherosclerosis [21,55] one study observed the opposite [22]. The basis for these conflicting findings is unclear. It might relate to the concomitant presence of diabetes, the type of feeding and the timing of the experiment. It is also unclear which cell type is important for the process. In general, smooth muscle cells are thought to exhibit the highest Nox1 expression in the vessel, but also endothelial cells and fibroblasts have Nox1 [5]. Outside of the vascular system, Nox1 has been shown to contribute to the inflammatory function of myeloid cells [56] and the liver [57,58], which is in line with the findings of the present work. Given that NoxO1 is an essential factor for Nox1-dependent ROS production [59], the present study to some extent supports a role for Nox1 in atherosclerosis development, although to our knowledge no gender bias has been reported for Nox1 so far. NoxO1 is also required for the activation of Nox3, but functional significant expression of Nox3 is restricted to the inner ear. In the case of overexpression, NoxO1 can also partially activate Nox2 [59], but our previous work found no evidence that a crosstalk occurs in vivo [11]. An alternative explanation is that NoxO1 has other functions than activating ROS production. For p47phox such functions have been reported [59,60]. Given the great similarity of NoxO1 to p47phox, an adapter function of NoxO1 beyond the one for Nox1-NoxA1 should not be dismissed.

Our study is limited by the fact that we failed to define the true mechanism of action of NoxO1 in the present model and that we did not study why the effect of NoxO1 deletion was restricted to the brachiocephalic artery and aortic arch in female mice. This was an active decision. The overall phenotype was weak and unfortunately, MACEseq was not direction giving for further mechanistic analysis. Thus, additional empirical testing would have been needed, in particular an excessive amount of animal work, involving models like ovarectomy and testosterone supplementation and tissue specific NoxO1 deletion as well as other models of atherosclerosis. Considering, the limited role of NoxO1 on a small aortic segment and gender-specificity, it appears unlikely that such an approach would provide the desired mechanistic insights.

In summary, the present study suggests NoxO1 to play a minor role in promoting atherosclerosis development in female mice, which is mediated by a reduced systemic inflammatory activity. Given that humans, compared to mice, have much lower levels of systemic inflammatory activity, it is unlikely that NoxO1 is an important target for the prevention of atherosclerosis.

## Source of funding

This work was supported by grants from the 10.13039/501100001659Deutsche Forschungsgemeinschaft (Auflösung von Entzündungen; GRK 2336, Sonderforschungsbereich 834 - Teilproject A2, excellence cluster EXS2026 Cardio-Pulmonary Institute) and by German Center for Cardiovascular Research (81X220014/81X2700221, and professorship of MS). HJ was supported by funding from the 10.13039/100000002National Institutes of Health grants HL119798 and HL095070.

## Declaration of competing interest

The authors declare that they have no relevant financial, personal or professional relationships to disclose which could be perceived as a conflict of interest or as potentially influencing or biasing the authors’ work.

## Abbreviations

EC: Endothelial cell
HDL: High density lipoprotein
LDL: Low density lipoprotein
Luc: Luciferase
MACE: Massive analysis of cDNA ends
NoxO1: NADPH oxidase organizer 1
PCSK9: Pro-protein convertase subtilisin/kexin type 9
ROS: Reactive oxygen species
SMC: Smooth muscle cell
TC: Total Cholesterol
TG: Triglycerides
