# Supplementary material for: Deletion of NoxO1 limits atherosclerosis development in female mice
Source: Redox Biol. 2020 Sep 4;37:101713. doi: 10.1016/j.redox.2020.101713 (PMC7502371; doi:10.1016/j.redox.2020.101713)
Supplement: Multimedia component 1 [file mmc1.pdf]

Supplementary data

A

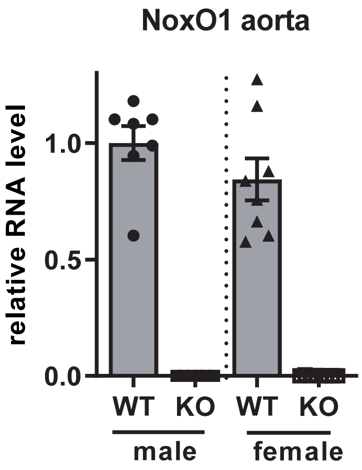

Supp. 1. Aortic Noxo1 mRNA level as determined by qRT-PCR

A) RNA was prepared from the thoracic aorta and NoxO1 expression was normalized to EEF2. NoxO1 primers were designed not to yield a transcript after NoxO1 knockout.

A

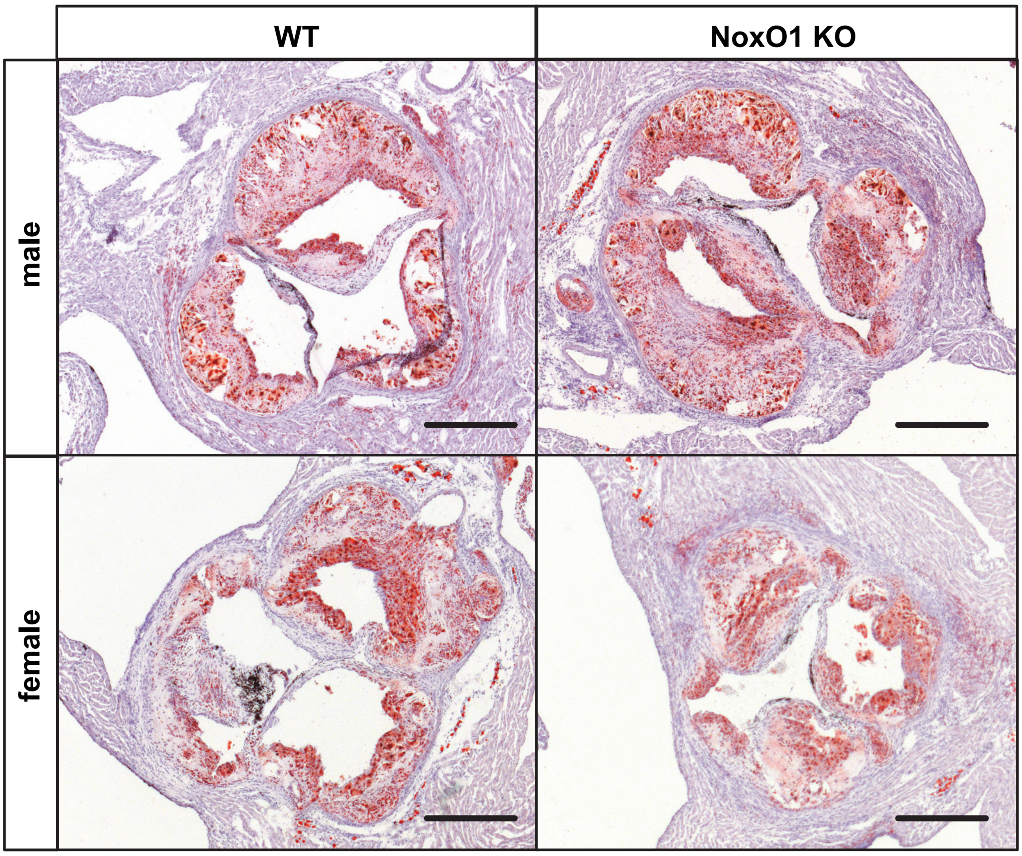

B

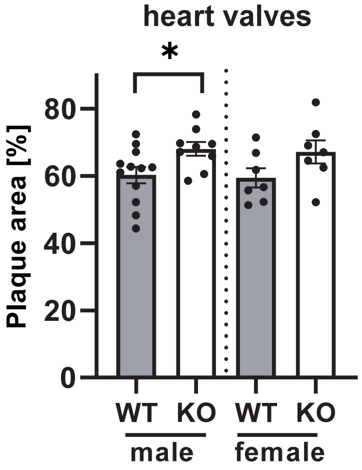

Supp. 2. Heart valves show immense plaque development
NoxO1 knockout (KO) and wildtype (WT) mice injected with AAV-PCSK9 and fed with Paigen diet for three month. **A** Heart valve plaque morphology of female and male wildtype and NoxO1 knockout mice (scale bars, 500µm). **B** Quantified heart valve plaque area (n=7-12; mean ± SEM, t-test; * p≤0.05).

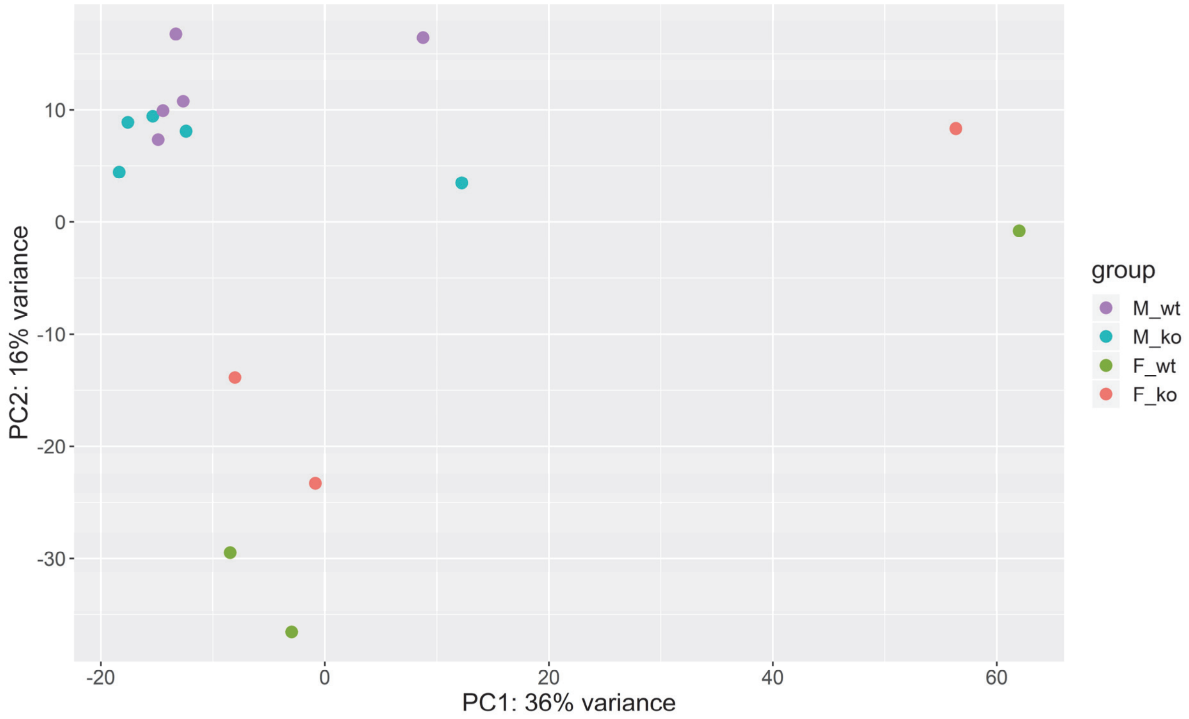

Supp. 3. Principal component analysis of MACE seq data
MACEseq dataset of wildtype and NoxO1 knockout abdominal aorta samples after AAV-PCSK9 treatment and 3 month Paigen diet. Principle component analysis of MACE dataset.

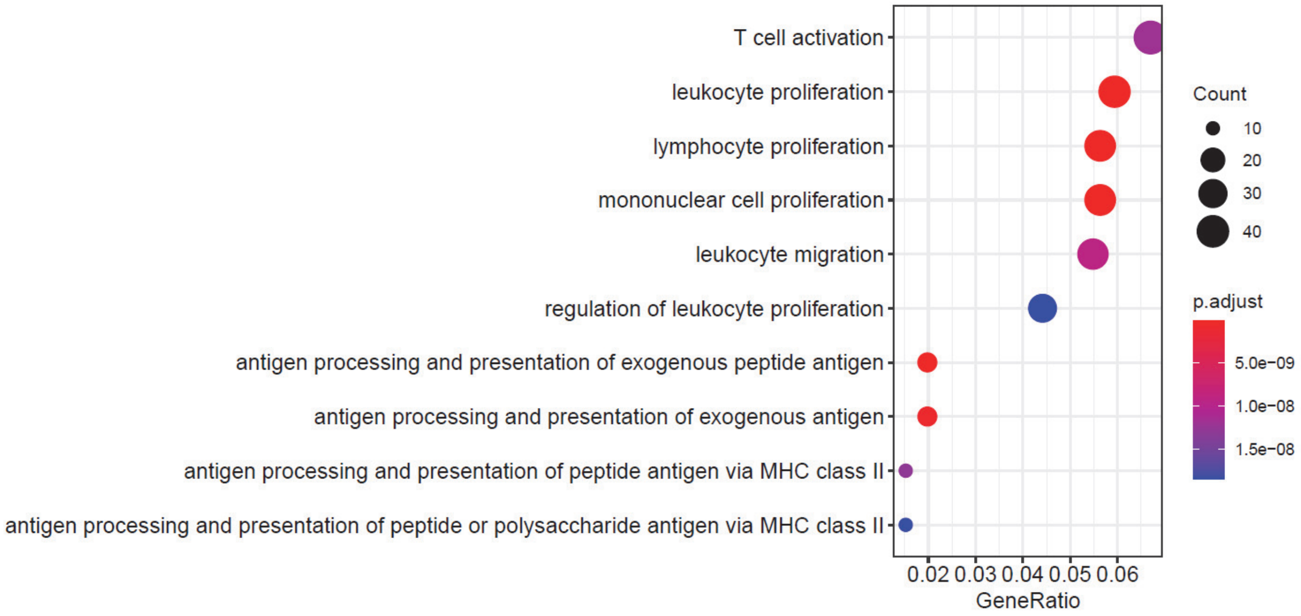

Supp. 4. GO term analysis of upregulated genes in female NoxO1 knockout

MACEseq dataset of wildtype and NoxO1 knockout abdominal aorta samples after AAV-PCSK9 treatment and 3 month Paigen diet. GO term analysis of upregulated genes comparing female KO versus WT.

A

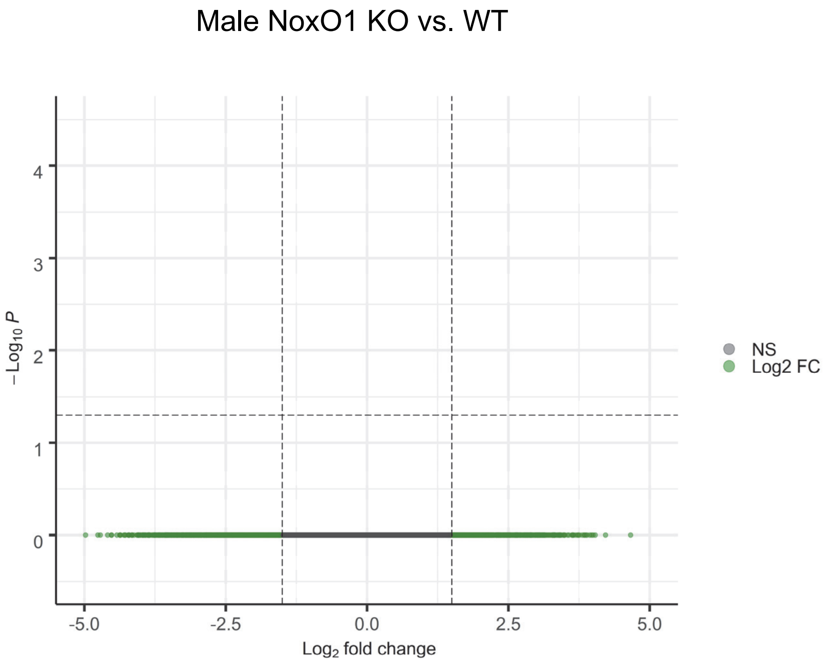

B

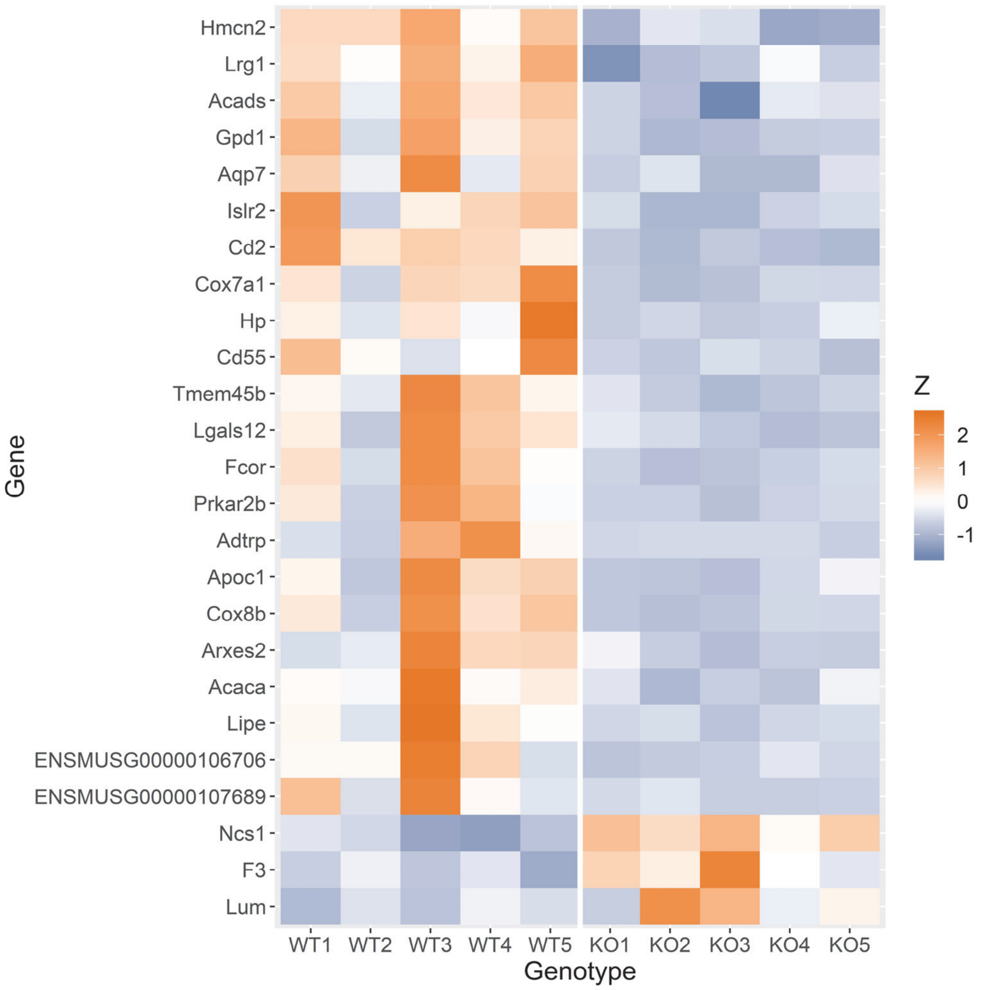

Supp. 5. MACEseq of males after NoxO1 deletion

MACEseq dataset of wildtype and NoxO1 knockout abdominal aorta samples after AAV-PCSK9 treatment and 3 month Paigen diet. **A** Volcano plot of male MACEseq comparing KO versus WT (FDR=0.05). **B** Heatmap of 25 most significant genes of male NoxO1 knockout compared to wildtype (n=5).

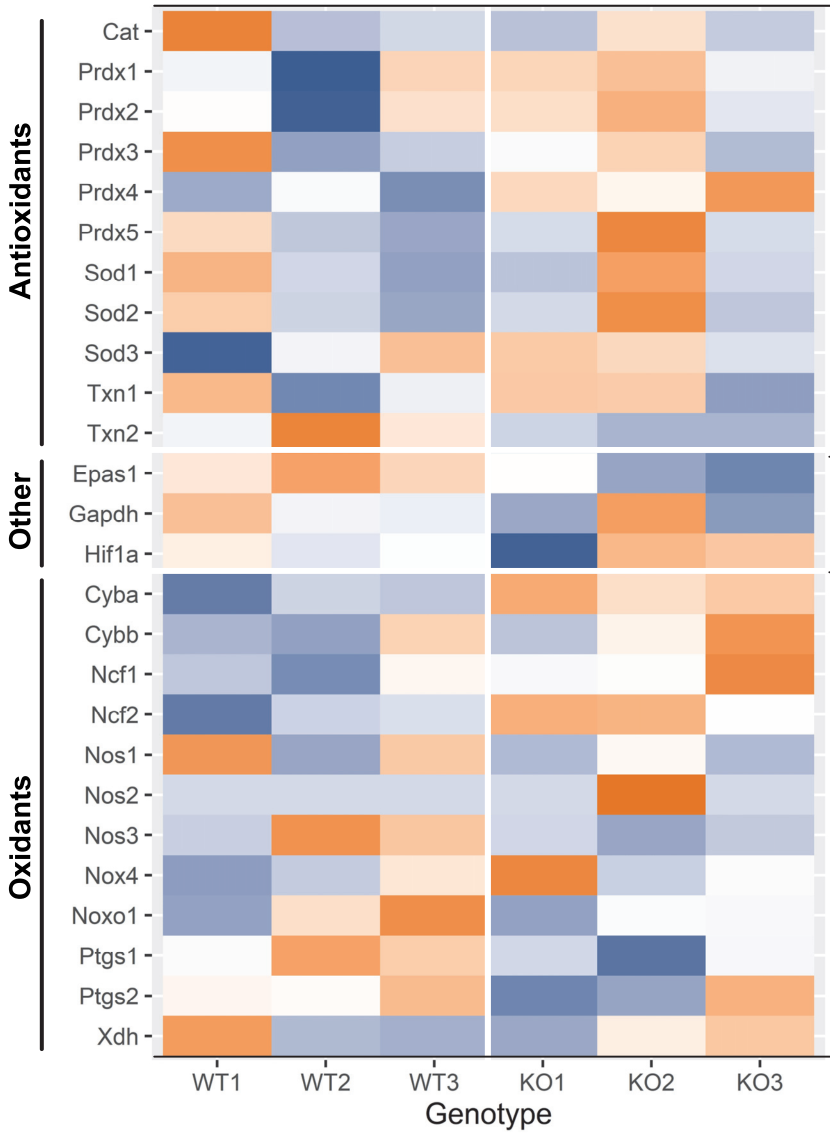

Mean counts WT	Mean counts KO	Gene
442	339	Cat
527	664	Prdx1
335	369	Prdx2
181	173	Prdx3
165	281	Prdx4
233	286	Prdx5
1145	1199	Sod1
148	95	Sod2
1465	1760	Sod3
469	518	Txn1
483	329	Txn2
1450	860	Epas1
140	126	Gapdh
197	197	Hif1a
267	465	Cyba
45	63	Cybb
21	36	Ncf1
19	32	Ncf2
9	3	Nos1
0	5	Nos2
296	156	Nos3
409	452	Nox4
7	3	Noxo1
206	123	Ptgs1
25	18	Ptgs2
106	111	Xdh

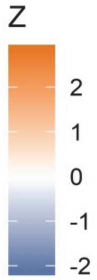

Supp. 6. ROS generators and antioxidants are not significantly changed by NoxO1 deletion in females

MACEseq dataset of NoxO1 knockout compared to wildtype abdominal aorta samples after AAV-PCSK9 treatment and 3 month Paigen diet. Genes involved in ROS generation and antioxidants. Female MACEseq samples comparing NoxO1 knockout versus wildtype (n=3, p=not significant). Please note that the knockout approach had no impact of the part of the sequence of NoxO1 covered by MACEseq. Therefore, it is expected that also NoxO1 expression could be determined.

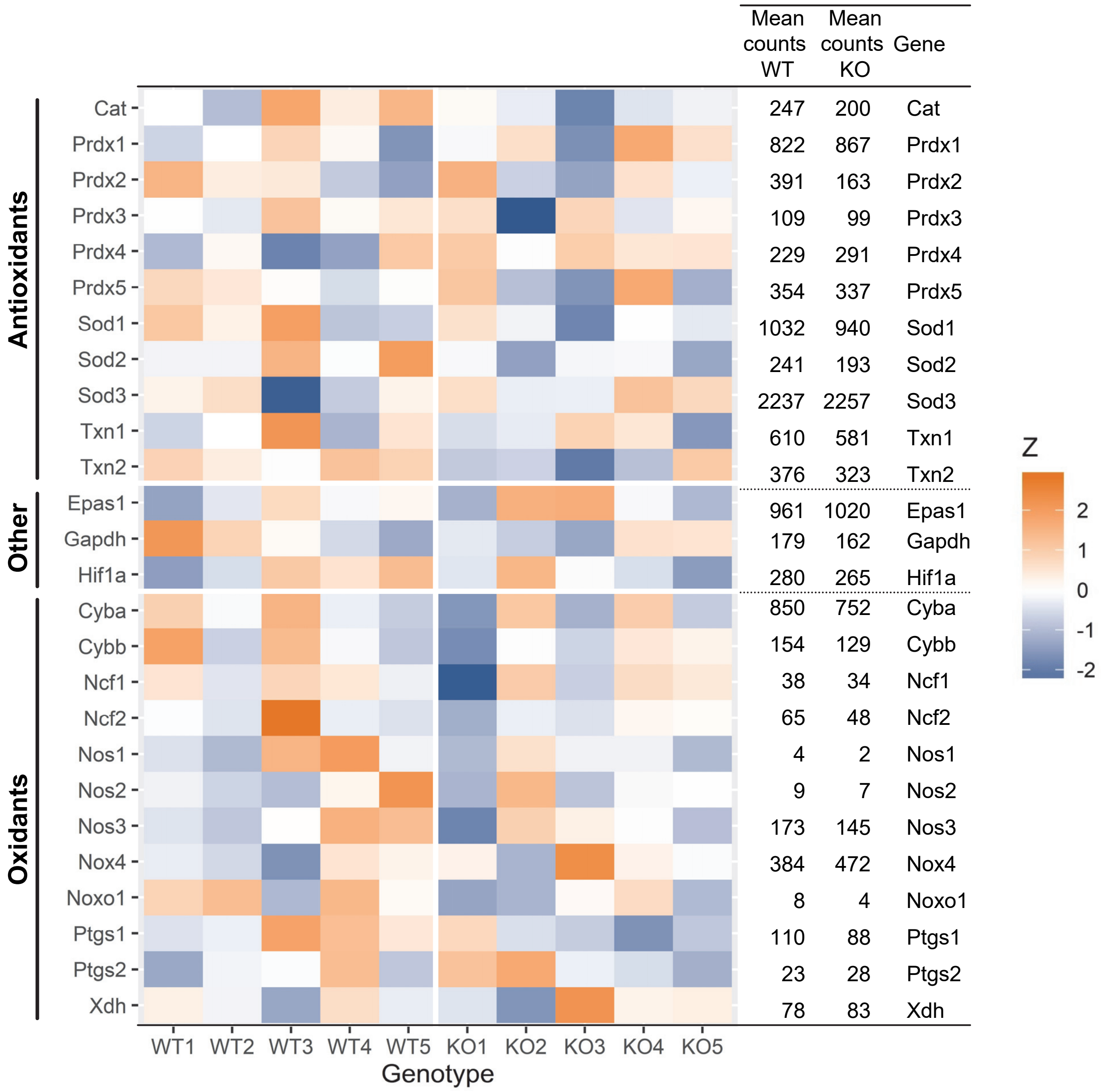

Supp. 7. ROS generators and antioxidants are not significantly changed by NoxO1 deletion in males

MACEseq dataset of NoxO1 knockout compared to wildtype abdominal aorta samples after AAV-PCSK9 treatment and 3 month Paigen diet. Genes involved in ROS generation and antioxidants. Male MACEseq samples comparing NoxO1 knockout versus wildtype (n=5, p=not significant). Please note that the knockout approach had no impact of the part of the sequence of NoxO1 covered by MACEseq. Therefore, it is expected that also NoxO1 expression could be determined.

A

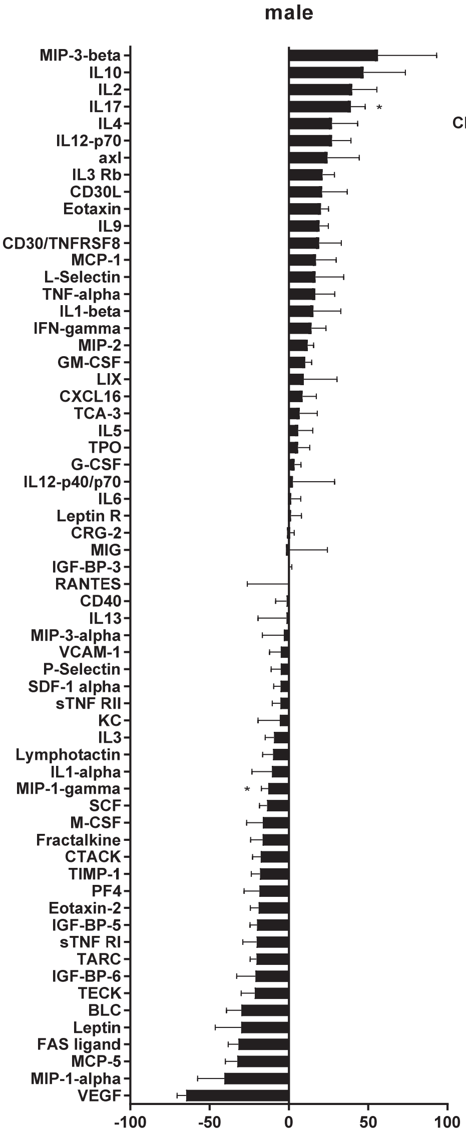

B

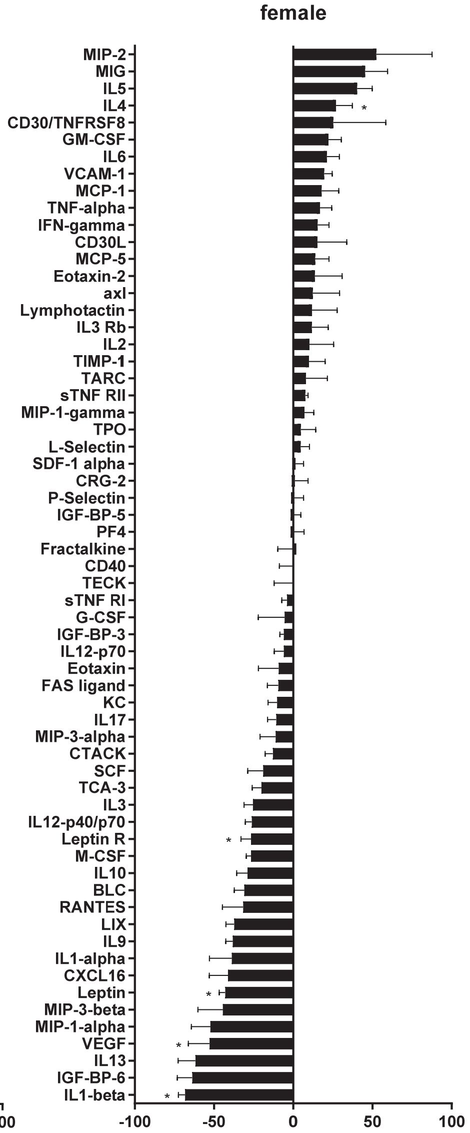

C

1. POS 1	36. IL-13
2. POS 2	37. IL-17
3. POS 3	38. KC
4. NEG	39. Leptin R
5. NEG	40. Leptin
6. Axl	41. LIX
7. BLC	42. L-Selectin
8. CD30 L	43. Lymphotactin
9. CD30	44. MCP1
10. CD40	45. MCP-5
11. CRG-2	46. M-CSF
12. CTACK	47. MIG
13. CXCL16	48. MIP-1a
14. Eotaxin	49. MIP-1g
15. Eotaxin-2	50. MIP-2
16. Fas Ligand	51. MIP-3 b
17. Fractalkine	52. MIP-3a
18. GCSF	53. PF-4
19. GM-CSF	54. P-Selectin
20. IFN g	55. RANTES
21. IGFBP-3	56. SCF
22. IGFBP-5	57. SDF-1a
23. IGFBP-6	58. TARC
24. IL-1a	59. TCA-3
25. IL-1 beta	60. TECK
26. IL-2	61. TIMP-1
27. IL-3	62. TNFa
28. IL-3 Rb	63. sTNF RI
29. IL-4	64. sTNF RII
30. IL-5	65. TPO
31. IL-6	66. VCAM-1
32. IL-9	67. VEGF
33. IL-10	68. NEG
34. IL-12 p40/p70	69. NEG
35. IL-12 p70	70. NEG

Supp. 8. Cytokine assay shows an anti-inflammatory signature in female NoxO1 knockout compared to wildtype mice

NoxO1 knockout (KO) and wildtype (WT) mice injected with AAV-PCSK9 and fed with Paigen diet for three month. **A** Cytokine assay analysis of male NoxO1 knockout vs. wildtype serum samples (mean ± SEM; n=4; t-test). **B** Cytokine assay analysis of female NoxO1 knockout vs. wildtype serum samples (mean ± SEM; n=4; t-test). **C)** RayBiotech AAM-CYT-G3 cytokine array panel.

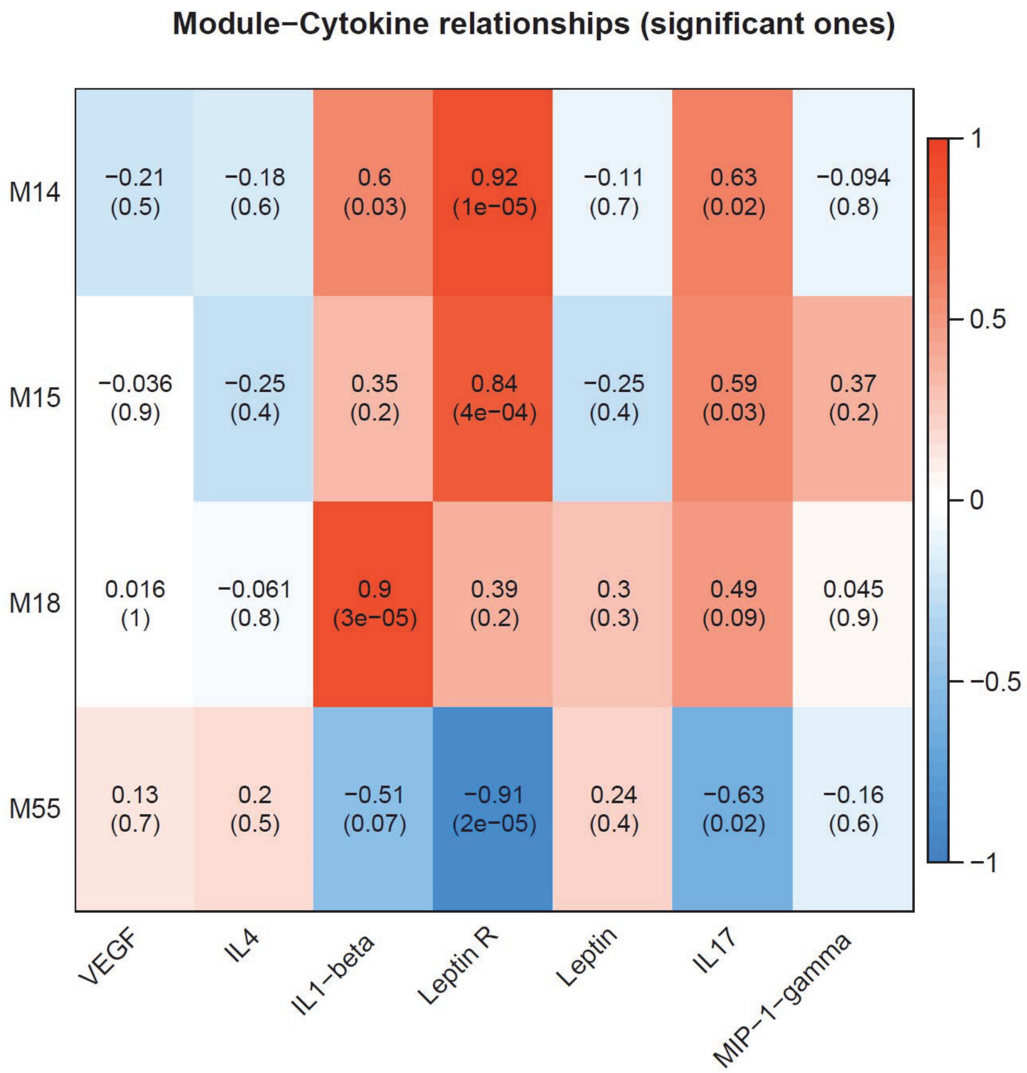

Supp. 9: Analysis of cytokine abundance and gene module expression correlation.
The mean expression of all genes in a co-expression module was correlated with the measured cytokine abundance (color scale, correlation). The correlation value and the respective p-value of that correlation value are written in each cell. The heatmap shows all gene modules for which an absolute correlation value of 0.8 and p-value < 0.001 was obtained for at least one of the significantly changed cytokines. Modules are shown in rows and cytokines in columns.

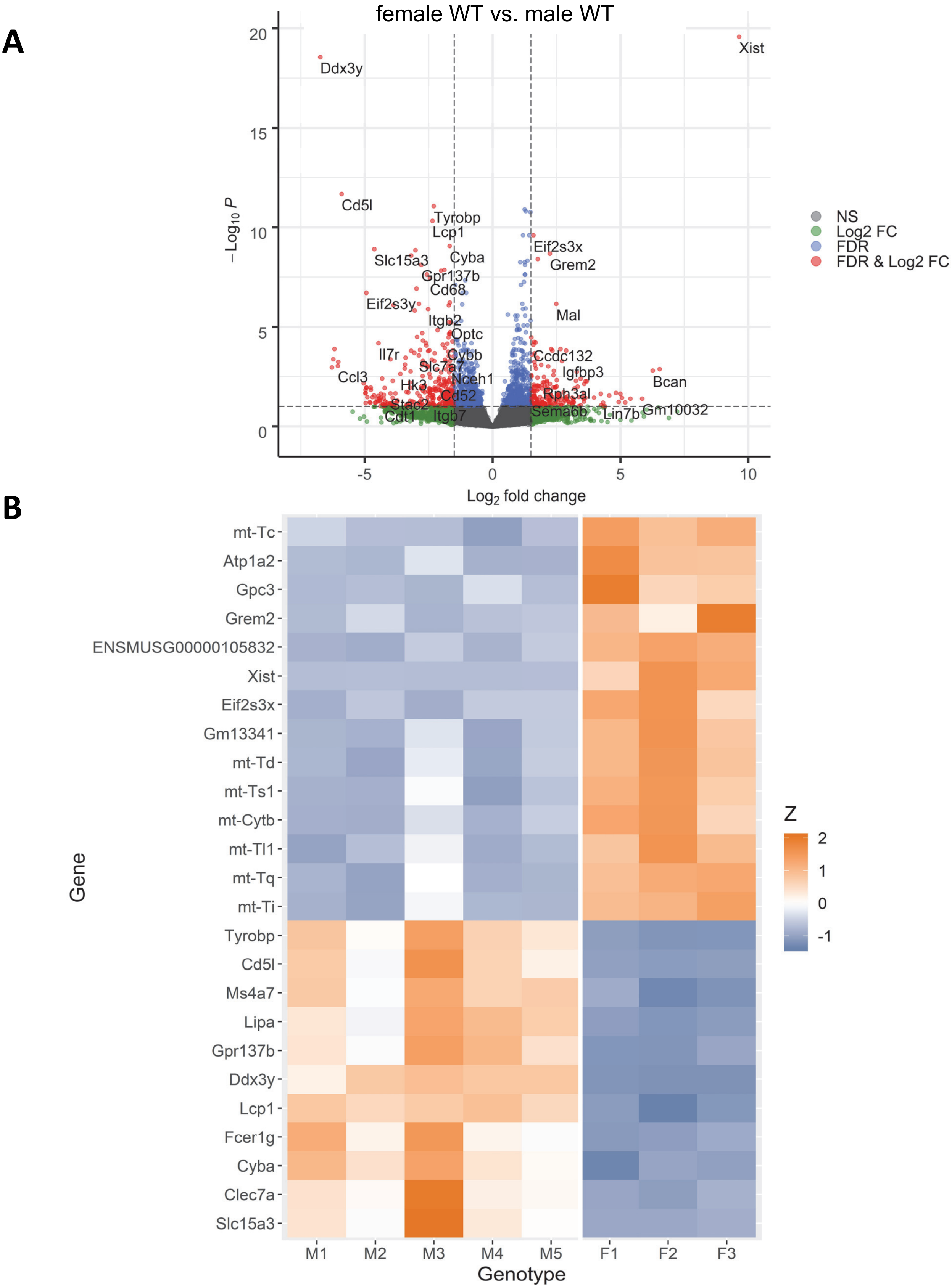

Supp. 10. DEGs comparing female against male wildtypes

MACEseq dataset of wildtype and NoxO1 knockout abdominal aorta samples after AAV-PCSK9 treatment and 3 month Paigen diet. **A** Volcano plot of MACEseq comparing female versus male wildtype (FDR = 0.05). **B** Heatmap of 25 most significant genes of wildtype female compared to male (n=3-5).
